# Discovery of metastases in thyroid cancer and "benign metastasizing goiter": a historical note

**DOI:** 10.3389/fendo.2024.1354750

**Published:** 2024-05-02

**Authors:** Sergiy Kushchayev, Yevgeniya Kushchayeva, Tetiana Glushko, Iryna Pestun, Oleg Teytelboym

**Affiliations:** ^1^ Department of Diagnostic Imaging and Interventional Radiology, Moffitt Cancer Center, Tampa, FL, United States; ^2^ Diabetes and Endocrinology Center, University of South Florida, Tampa, FL, United States; ^3^ Department of Radiology, Mercy Catholic Medical Center, Philadelphia, PA, United States

**Keywords:** cancer, thyroid, metastases, malignancies, goiter, history

## Abstract

At the beginning of the eighteenth century, most physicians recognized cancer as an aggressive process that gradually spreads, leading to cachexia and death. Thyroid malignancies had long been underestimated because the majority of the population of West Europe suffered from diffuse goiters that masked malignant processes in the neck. Moreover, the life expectancy at that time was very low (about 37-40 years), so the majority of people died of other causes before metastatic thyroid cancer could develop and manifest. Nevertheless, in 1817, French dermatologist Jean Louis Alibert described the first case of a malignant tumor involving the thyroid gland. From the 1820s the number of case reports describing thyroid cancer increased. Even though Jean Claude Recamier described *metastases* in 1829, secondary lesions on various organs in patients with thyroid malignancies were not themselves considered malignant until 1876.

In 1876, Max Runge and Julius Cohnheim independently published cases of thyroid carcinomas with metastases. The famous German pathologist Von Recklinghausen confirmed the malignant etiology of the secondary tumors in both cases. Both publications brought attention to the problem of thyroid cancer, resulting in a fruitful discussion in the scientific literature. However, despite this apparent evidence of malignancy, Julius Cohnheim introduced the concept of *benign metastasizing goiter.* This theory remained popular in medical society for a long time. It required multiple studies to prove that the presence of metastases is a crucial indication of thyroid malignancy.

In the nineteenth century and beginning of the twentieth century, the physiologic *function* of the *thyroid gland* was unknown. There were several observations that led to the recognition that the thyroid is an endocrine organ. In 1874 British William Gull was the first to describe myxedema what he called a “cretinous state in the adult” (1, 2). In 1878 William Ord from St. Thomas Hospital in London published a paper in which he coined the term myxedema and published the first photography of a patient (2, 3). In 1882 Jacques-Louis Reverdin with his cousin and assistant Auguste in Geneva, described “*myxoedème opératoire*” in subjects upon which fatal thyroidectomies had been performed, and Theodor Kocher, in the following year, reported similar results in operative cases, but termed the condition “*cachexia strumipriva*,” *(* 4, 5). Both Reverdin and Kocher contributed to the discovery that lack of the thyroid gland causes severe physical and mental damage (6). In a paper entitled “On the Function of the Thyroid Gland,” published in 1884, Sir Victor Horsley wrote that before Theodor Kocher’s publications in the 1880s, were three main theories concerning function of the thyroid gland: “*1… that the thyroid body acted as a regulator of the circulation in the brain, and possibly manufactured some substance which was of primary importance for the nutrition of the central nervous system. 2. That it was a true gland, and secreted a mucinous albuminoid into the cavities of its acini… 3. The thyroid gland has also from time to time been compared to the spleen as an haemapoietic organ” *(7).

During the first half of the nineteenth century, most physicians recognized cancer as an aggressive process that gradually spreads, leading to cachexia and death if left untreated (8). Pathologists used the newly invented *microscope* to diagnose tumors, and subsequently, the microscopic diagnosis of tumors became routine. Although enlargements of the thyroid gland had been known for some time, it was not until the beginning of the nineteenth century that malignant tumors were discovered. At that time, many of the residents of Western Europe suffered from iodine deficiency and thus diffuse goiters that masked malignant processes in the neck. Life expectancy was also very low at that time, about 37-40 years (9), so the majority of people died from other causes before metastatic thyroid cancer could develop.

In 1817, French dermatologist Jean Louis Alibert, in his book “*Nosologie naturelle ou les maladies du corps humain: distribuées par familles: Caille et Ravier”*, became the first to describe a case of malignant tumor involving the thyroid gland, which he termed a “*sarcoma of the thyroid body*” (10–12) (**Figure 1**). From the 1820s the number of case reports describing thyroid cancer increased. In 1823, British physician Allan Burns also reported a case of thyroid carcinoma in his book “*Observations on the surgical anatomy of the head and neck: illustrated by cases and engravings”* (13). Burns described a woman who died of dyspnea and dysphagia secondary to a “*medullary sarcoma of the thyroid*,” which was “*big as the first … and hard as a stone*” (14). In 1833, Gaspard Laurent Bayle, in his “*Traité des maladies cancéreuses”*, also described a case of thyroid cancer and noted that thyroid cancer was rare and could be either secondary or primary (15). Later, in 1838, French physician Auguste Gabriel Maurice Raynaud, in a publication entitled “*Thyroscarcomie*,” reported an “*encephaloid sarcoma*” with metastatic foci in the lungs and liver, but he unfortunately provided no description of the microscopic findings (10) and did not use the term metastasis. Working at Massachusetts General Hospital, famous American surgeon John Collins Warren also described a *“scirrhous carcinoma of the thyroid”* (13, 16). Rudolf Virchow states that he saw one *“sarcoma of the thyroid gland”* and mentioned malignant tumors of the thyroid in his publications, but provided no data regarding their occurrence and no descriptions of the tumors themselves (10, 17). At that time, the exact differences between different types of malignant tumors were unknown.

**Figure 1:**
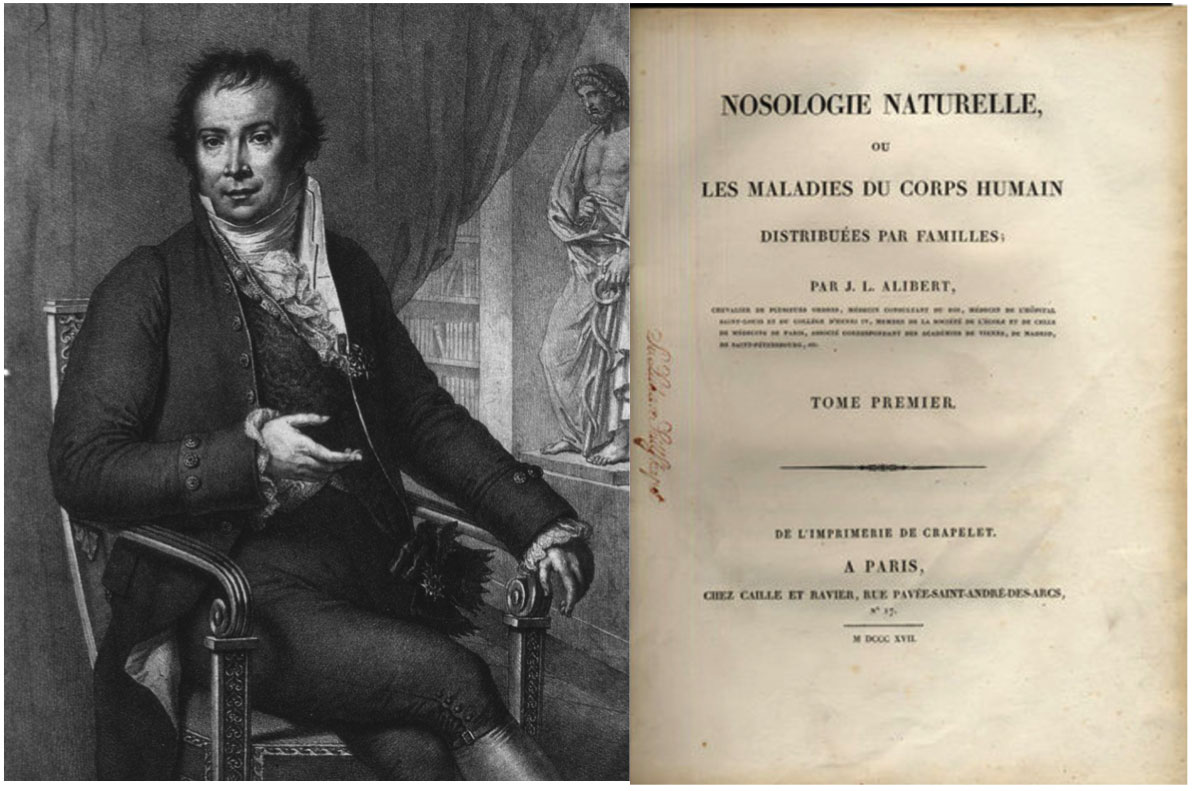
Jean-Louis-Marc Alibert (1768 – 1837), a French dermatologist, who was the firs to describe “*sarcoma of the thyroid body*”. The image was taken from an open source: https://en.wikipedia.org/wiki/Jean-Louis-Marc_Alibert. The Alibert’s book entitled “*Nosologie naturelle ou les maladies du corps humain: distribuées par familles: Caille et Ravier*”.

Using the same approach as that used with solid tumors in other locations, pathologists eventually came to recognize neoplastic cells within the thyroid gland and use more specific terminology. At least in books published between 1840 and 1850 in Germany and the UK, thyroid cancer was described as a defined entity. For example, in 1846, British Walshe, in his book “*The Nature and Treatment of Cancer”*, wrote the following: *“The thyroid gland is very rarely the seat of cancerous disease; nevertheless, cases are recorded in sufficient number to exhibit its characters, both anatomical and symptomatic, under very various conditions”… “Eight of 8289 deaths from cancer in Paris are ascribed to the thyroid gland” (* 18).

Thyroid malignancies were rarely diagnosed at that time; only clinically advanced cases of thyroid carcinomas were recognized. The majority of localized forms of thyroid carcinoma had never been reported. In 1844, Robert Liston, emphasized, that “*it is desirable that you should be able to distinguish the swelling of the thyroid body from the other tumors that occur in the neck.”* He proposed that thyroid swelling can be diagnosed *“by placing your fingers over the prominent part of the thyroid body, and then, desiring the patient to swallow his saliva, you find that it moves rapidly upwards under your fingers, and there is then no any doubt about the case*” (19).

Interestingly, at that time, thyroid cancer was considered to be a very aggressive form of malignancy. *“When primary, the disease may prove fatal, before any other organ becomes implicated; in other words, it continues from first to last solitary”* (18). “*Most cases of malignant disease develop in a preexisting goiter in patients from 40 to 60 years of age.” *(20) The pathologic entities were certainly distinct from the situation observed today. It is well known that follicular thyroid cancer is more prevalent under conditions of insufficient iodine intake and probably was more prevalent than nowadays. Indirect support of this hypothesis supports the fact of the high frequency of bone metastases among patients with thyroid cancer (21). *The prognosis in this disease is bad. The average duration of life in cancer of thyroid is … only six month … It has been estimated that 60 per cent of the operated cases die in eight weeks and 84 percent in six weeks*” (20).

Although there was no thyroid carcinoma classification at that time, many divided thyroid carcinoma cases into tuberous and infiltrative forms: *“In the tuberous form the disease displays little or no tendency to encroach upon neighbouring parts; in the infiltrated form (whether the infiltration have originated in the proper substance of the gland or not) such tendency is invariably observed, and in its consequences commonly proves destructive of life”* (18)*. “Encephaloid and scirrhus, simple and of the mastoid, solanoid, and haematoid varieties” *(18) of thyroid tumors were also reported. However, it is difficult to project this terminology onto modern thyroid cancer classifications. Certainly, terms and nomenclature used to describe the findings at that time had a different meaning than that understood today.

The majority of the patients with thyroid malignancies presented with airway compression and dysphagia, but unfortunately, the surgical treatment of thyroid diseases was rare even in advanced cases due to the risk of uncontrolled bleeding. *“The dangers attending the extirpation of the thyroid gland, merely considered as a surgical operation, are almost proverbial; it seems difficult to conceive the attempt to remove the organ, when affected with cancerous disease, is anywise warrantable” *(18).

At the end of nineteenth century, Theodor Kocher revolutionized thyroid surgery, and total thyroidectomies became a means of treating endemic goiters and in a lesser extend, thyroid malignancies. However, due to *“cachexia strumipriva”* (by Theodor Kocher, 1884) or “*myxoedème opératoire*” (by Jacques-Louis and Auguste Reverdin, 1882), which is known as postsurgical hypothyroidism, initially surgery was not used that frequently (22). *“There are no remedies which possess any power to arrest the progress of thyroid carcinoma. Whenever symptoms of suffocation appear, the diseased mass should be removed merely as a palliative measure. The results of such operations have been noticed in connection with the subject of extirpation of the thyroid gland” *(23). Because there was no cure, Kocher limited his practice to one-sided lobectomy, reserving total resection for cases of intractable compression or cancer (24). In 1907, Kocher reported the occurrence of about 400 cases of malignancy in something over 4,500 operated goiters. He claimed that such diagnoses were made too late, though with a little more searching, care and understanding, such situations could be easily controlled (11).

The term “metastasis” was introduced in 1829 by Jean Claude Recamier to describe “*the transfer of disease from one organ or part to another not directly connected to it*” (25). Metastases originating from other non-thyroid primary location were widely reported in the literature. However, metastases arising from thyroid carcinoma were not scientifically established until 1876 (26, 27).

Several authors described findings consistent with thyroid cancer metastases but either did not believe that those metastases were related to primary thyroid carcinoma or did not paid sufficient attention to these findings. In 1843, British scientist Hawkins published a case of thyroid carcinoma with lesions that according to our modern knowledge, may represent parenchymal metastases: *“The liver contained many tubercles of the same character as those found in the lungs; they were small, none larger than a filbert, and not softened.” *(28) In 1845, the American Warren published a case of a 25-year-old painter who had a *“tumour on the right side of the neck: it had existed for a year and a half.” *(16). Unfortunately, the patient died. *“Among the post-mortem appearances were the following: The surface of the body was studded with subcutaneous tumours, composed of small granulations, constituted by sacs containing a substance which appeared at first view to be wholly gelatinous, but which, on being divided, discharged a small quantity of viscid fluid….Similar tumours existed in the thyroid gland, diploe, muscles, mediastinum, muscular substance and cavities of the heart, lungs, pancreas, kidneys and testes … The liver contained scirrhous nodules; the absorbent vessels of the surface of the abdomen contained a matter having some of the characters of encephaloid.”* (29). Findings consistent with skin thyroid metastases were also described in 1845 as “*hard tubercules … composed of the same substance as main disease mass which occupied the left lobe of the thyroid. This lobe was somewhat enlarged and converted to the mass of carcinomatous structure white, hard as a cartilage, and with some gritty particles dispersed through it* (28, 30). In 1867, the British Moxon also described a case of thyroid malignancy with multiple lesions throughout the body, including the lungs, heart, and pericardium, which may also represent metastatic disease (31).

However, the recognition of metastatic disease related to thyroid carcinoma begin in March of 1876, when German obstetrician and gynecologist Max Runge published the first case of confirmed metastases arising from the thyroid gland in “*Virchow’s Archiv für pathologische Anatomie und Physiologie und für klinische Medicin”* (26). He presented the case of a 41-year-old pregnant woman with metastases to the C1 and C2 vertebrae, who died secondary to spinal cord compression in August of 1875. Six months later, in October of 1876, in the same journal, another German pathologist, Julius Cohnheim, reported a case of metastastic thyroid tumor that occurred in December of 1875. Both cases occurred during approximately the same period of time and contributed immensely to furthering our understanding of thyroid cancer. We are pleased to present these cases in this article.

## Case #1. Metastasis to epistropheus, Dr. Runge’s case

**Figure 2:**
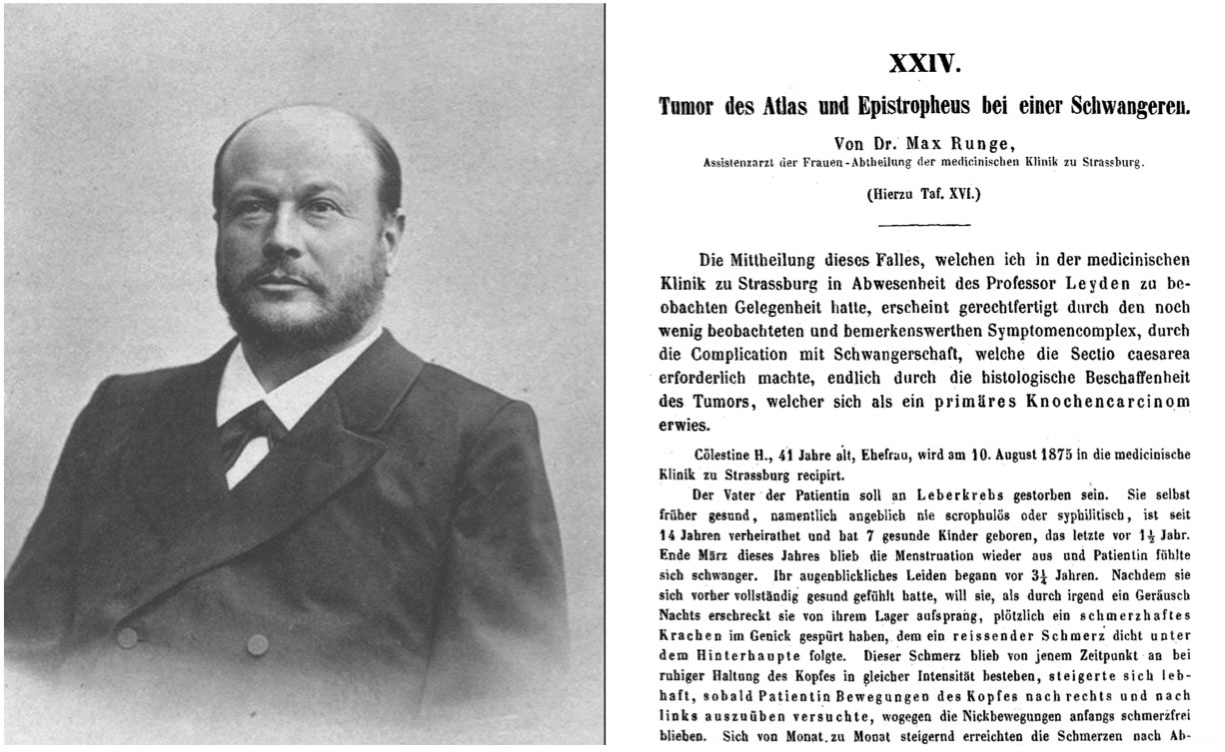
Heinrich Max Runge (1849– 1909), a German obstetrician and gynecologist who was the first to describe a histologically proven case of spinal metastases secondary to thyroid cancer.The image was taken from an open source: https://en.wikipedia.org/wiki/Max_Runge#/media/File: Runge,_Max_Heinrich.jpg. The first page of the article entitled “Tumor des Atlas und Epistropheus bei einer Schwangeren”. The paper was published in “*Archiv für pathologische Anatomie und Physiologie und für klinische Medicin*” in March 1876 (26).

Max Runge described a case of what he believed was primary bone cancer with *“little-observed and remarkable complex of symptoms, by the complication of pregnancy”* in a 41-year-old woman (**Figure 2**). She was admitted to the medical clinic at Strasbourg on August 10, 1875. The patient died the next day, and the premature child she delivered by C-section died two days later.

Her complaints started three and a quarter years prior to her admission when *“she suddenly felt a painful crack in the neck…*.” The patient developed weakness in her right arm and leg, which progressed to complete paralysis with persistent pain nine months prior to admission. The patient also reported difficulty swallowing solid food; however, her facial nerves, language, and cognitive abilities were not impaired. On admission, the patient was not able to move her head voluntarily. She was not cyanotic, with a weak pulse of 120 bpm. Her body temperature was 38.7 C.

Runge continued to describe the patient as follows: *“The head slackly hangs down to the left side … The left pupil is considerably wider than the right pupil; both react sluggishly … Active turning of the head is impossible. If you take the head into your hands, you can turn it passively without much resistance from the musculature … If her head is strongly bent forward, the patient gets a seizure of dyspnea, in which the eyes stand out and the face turns bluish.”* Complete paralysis of both arms and the right leg without muscle wasting or contractures was noted. *“Strong pressure in the area of the first cervical vertebrae especially to the right of the median line of the neck produces pain.”* The neck showed thyromegaly, especially of the right side.

Runge concluded as follows: *“After this finding and anamnestic data, the diagnosis was made for a disease of the upper cervical vertebrae, which led to a compression myelitis of the upper cervical medulla. According to the anamnesis, the symptoms of an affection of the first two cervical vertebrae arose, which was followed by hemiplegia, then considerable motor paraplegia.”* Based on the symptoms, the first atlas (C1) and axis (C2) (“Epistropheus” is an old name for the C2 vertebra) of the cervical spine were affected.

The night after admission, *“the patient spends a sleepless night because of more severe pain in the extremities … Around 10 o’clock in the evening the respiration suddenly becomes irregular and the patient is cold”… “At 10:40, the mother’s last heart sound disappeared, and the immediate operation brought to light a seemingly dead male child who was recalled to life after about 10-15 minutes”. “On the second day after surgery it became abnormally drowsy and cool and died about 48 hours after surgery”.* The patients died.

**Figure 3:**
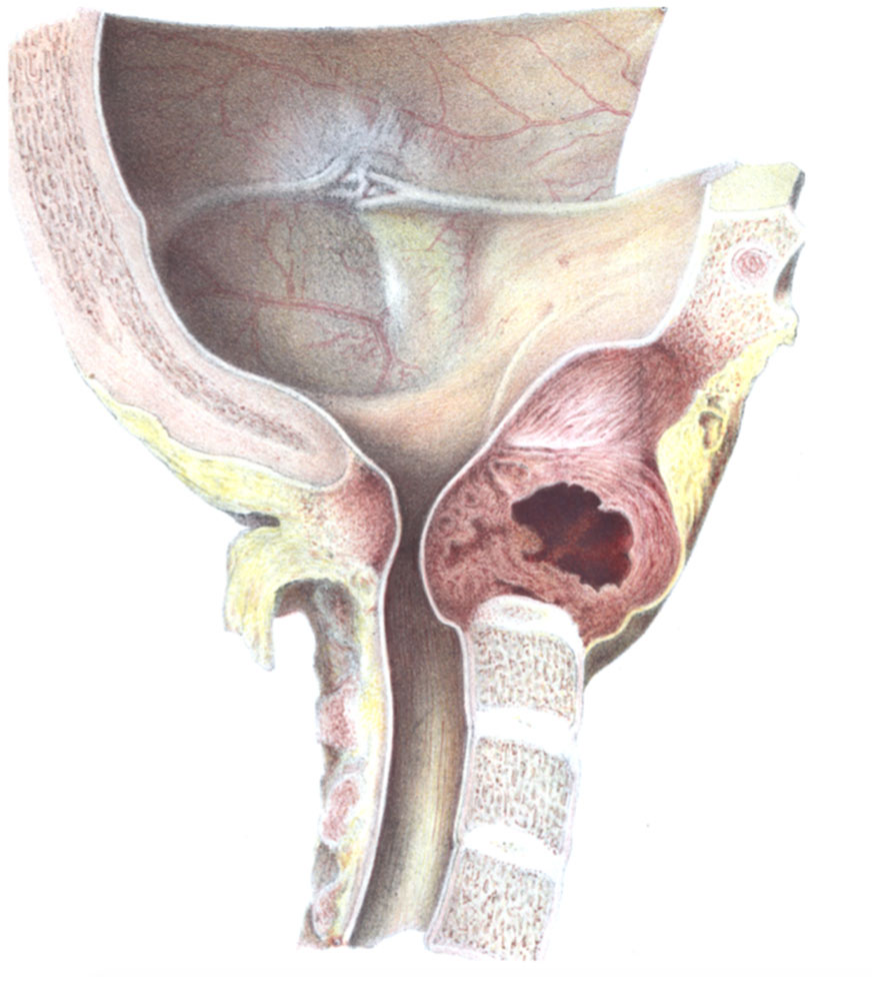
The original illustration of the metastatic tumor involving the C1 and C2 vertebrae secondary to thyroid carcinoma, published by Max Runge in 1876 (26).

An autopsy was performed by another German pathologist Zahn: “*On the median section of the hardened preparation through the cranial base and cervical spine it is revealed that the tumor is formed at the site of the atlas and epistropheus and, furthermore, occupies the right side of the occipital bone and the clivus approximately up to the no longer detectable sutura sphenooccipitalis … A closer examination of the spinal cord revealed a rather horizontal insertion from the front underneath the medulla oblongata, which became very clearly marked after hardening. The substance showed the approaching alterations of myelitis compression, … The thyroid is strongly enlarged, containing several adenomas with a connective tissue capsule. Microscopic examination of the thyroid does not yield anything suspicious.”* (**Figure 3**).

The famous German pathologist von Recklinghausen described the following microscopic changes: *Microscopic sections shows almost everywhere the same construction, namely, 1) a scaffolding substance, 2) clusters and strands stored therein that are formed from cells … The cells themselves then appear somewhat flat, are small, so that there is a certain similarity with thyroid tissue, in which early colloidal degeneration is present. Since clusters of cells, which are similar to epithelial cells, stored in alveoli between a connective tissue scaffolding substance are formed, the diagnosis must be made to carcinoma”*.

## Case #2. “Benign” metastasizing goiter of Julius Cohnheim

**Figure 4:**
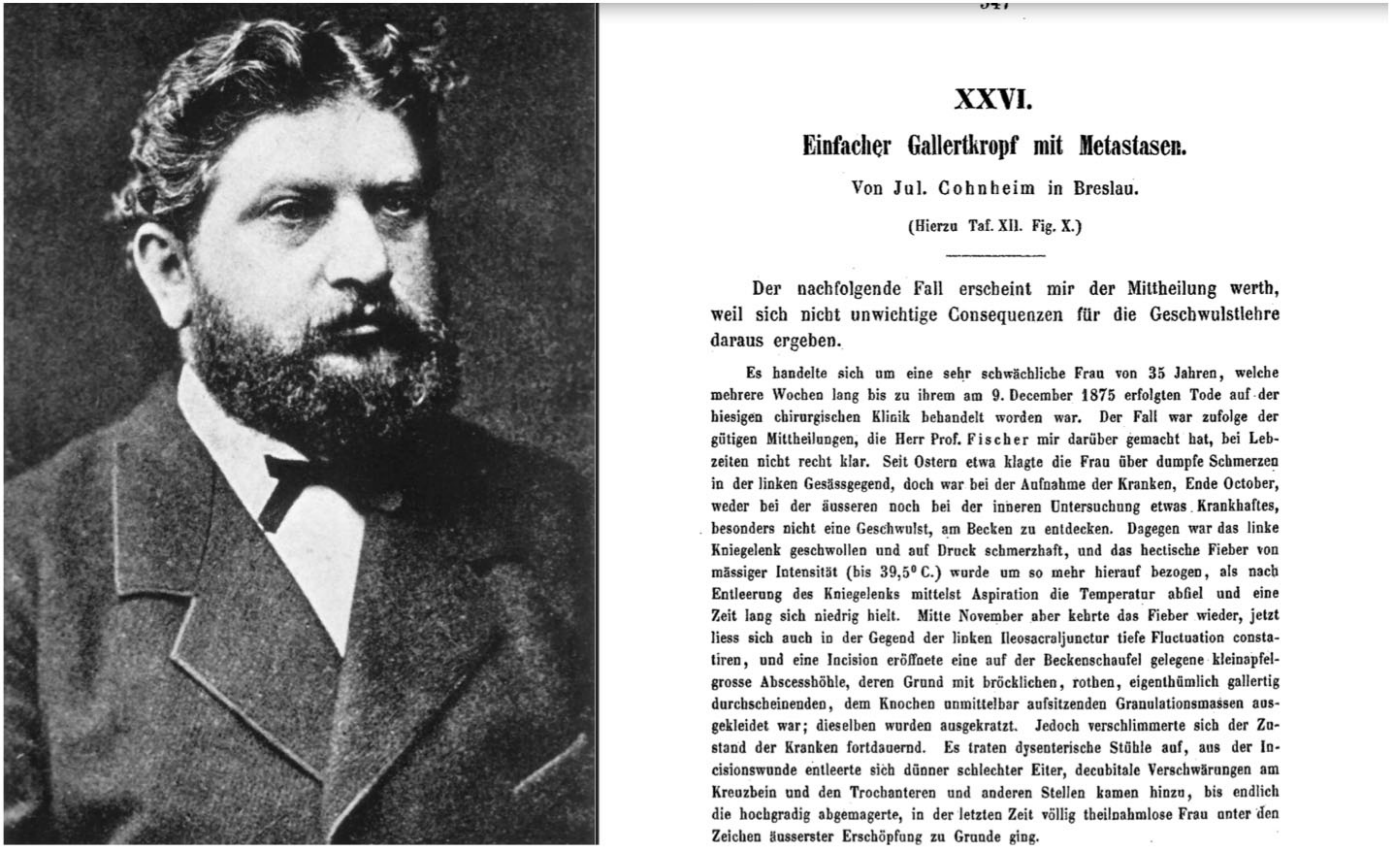
Julius Friedrich Cohnheim (1839 – 1884), a German pathologist who published the case of “ The image was taken from open source: https://en.wikipedia.org/wiki/Julius_Friedrich_Cohnheim. The first page of the article entitled “*Einfacher Gallertkropf mit Metastasen*” published in “*Archiv für pathologische Anatomie und Physiologie und für klinische Medicin*” in October 1876.

In October 1876, Julius Cohnheim, from Breslau, published a paper entitled “*Simple Gelatinous Goiter with Metastasis.*” (**Figure 4**). He wrote of “*A woman of 35 years who had been treated for several weeks until her death on December 9, 1875… The woman complained of a dull pain in the left region of the buttocks … On the other hand, the left knee joint was swollen and painful under pressure, and a hectic fever of moderate intensity (up to 39.5°C) was even more related to this. After emptying the knee joint via aspiration, the patient’s temperature decreased … However, in the middle of November, the fever returned; now, in the region of the left ileosacral junction, a deep fluctuation was observed. An incision opened a small-apple-sized cavity in the abscess, whose base was lined with crumbly, red, peculiarly gelatinous, translucent granular masses that were directly attached to the bone; those masses were scraped away. However, the condition of the patient continued to worsen.”* Ultimately, the patient died.

Cohnheim performed the autopsy and noted “*the extreme emaciation of the whole body, brown atrophy of the heart, and extensive diphtheria of the colon*. *There was an abscess on the external surface of the left ilium up to the symphysis, which was completely ulcerated, with the total loss of the cartilage coating and superficial caries on the articulation surfaces. On the ilia bones, at the site of the abscess, only shallow caries could be recorded.”* Based on the description, the author is describing a large bone metastasis causing skin ulceration.

Then, Cohnheim provided a very detailed description of the pulmonary metastases. “*First, about the lungs … small nodules were felt on the surface and inside both lungs. As a matter of fact, as they appeared on the cross-section, they were interspersed with a very large number of pinhead-sized to pea-sized soft knots, all of which were sharply differentiated from the surrounding tissue … they were all distinctly translucent, so they immediately brought to mind, at first sight, a struma gelatinosa.”*.

Then, the author describes the thyroid: “*Both lobes of the thyroid gland are enlarged, the left more so than the right. The right side of the tissue shows only the ordinary follicular, granular structure, while there are two larger nodules on the left, the smaller of which is far down behind the manubrium sterni. The cross-section shows behavior quite typical of a gelatinous goiter, and adds to the confusion regarding the left-sided bronchial glands, especially the upper, pigeon-egg-sized nodule. A third, pea-sized gelatinous nodule sits apart from the main mass of the thyroid gland to the upper left of the thyroid cartilage, somewhat displaced. In other respects, the thyroid gland absolutely does not offer anything conspicuous; the external configurations show a deviation from the norm caused by a moderate enlargement and the goiter nodules….In the finer preparation, it is still evident that the lower, gelatinous node with a small, smooth, knob-shaped bulge extends into the lumen of a larger, inferior thyroid vein*”.

Spinal metastases were also found: *“The second, third, and fourth lumbar vertebral bodies were found to be riddled with reddish, quite raspberry-jelly-like masses.*” Metastasis to the right femur was also described: *“In addition, in the medullary cavity of the right thigh bone, I came across a smooth hazelnut-sized node articulated sharply against the environment. This node was of orange-red honey-like appearance. Thus, from within, the cortex was a little indistinct, so the medullary canal, at this point, featured a flat bulge nearly 3 cm long.”*.

Describing the microscopic findings, the author stressed the following: “*I was not a little astonished because the first microscopic section from one of the lung metastases presented the complete and typical picture of the thyroid tissue…”* Similar changes were found in the other organs*: “The tumors resembled each other so much that it was practically impossible to distinguish microscopic sections of the lung nodes, the thyroid gland, and the bones from one another*”.

At the end of the paper, Cohnheim summarizes his findings and draws a conclusion: “*that the simple gelatinous adenoma that had formed in the thyroid gland in this woman created metastases in other organs, namely the lungs, lymph glands, and bones*.” In this case, Cohnheim underestimated the invasion of the lumen of the left inferior thyroid vein and explained the presence or absence of metastases in such cases of venous invasion by assuming a special constitutional individuality (32): “*Also, I would like to point out that in our case, the proven ingrowth of a goiter nodule into a vein does not have great importance, because this occurs fairly commonly in tumors of all kinds. In any case, even in the case of the presumed abrasion of the tumor particles through the blood stream, this is not sufficient to explain the development of metastatic nodules in remote locations.”*.

Moreover, Cohnheim states that the Max Runge’s case was indeed another representation of benign thyroid tumor with metastasis: *“I am now of the opinion that this atlas tumor certainly existed for 3 1/4 years and that in the severely enlarged thyroid gland, several adenomas were identified during the sectioning, so it does not seem to me too daring to interpret the case as a gelatinous goiter with metastasis in vertebral bodies.”*.

Cohnheim believed tumors in such cases to be benign, but von Recklinghausen and Wölfler disagreed with him and held that the findings indicated malignancy, claiming that a tumor capable of giving rise to metastases was not entitled to the denomination of benign (33). In opposition to Cohnheim, von Recklinghausen believed that the tumor in question was a “jelly carcinoma” and even published an article with illustrations in which he insisted on the malignance of the tumor (34). In a paper on this matter entitled “*Bemerkungen zu dem Aufsatz des Herrn Cohnheim: Einfacher Gallertkropf mit Metastasen*,” von Recklinghausen not only showed that the specimens represented malignancy but also, for the first time, that the metastases in the bone were the result of malignant emboli in the marrow capillaries (34, 35). Another prominent physician of the time, Billroth’s pupil Anton Wölfler, who was the first to describe postoperative hypoparathyroidism, also believed that metastatic spread indicated an increased proliferative energy, which is one of the chief characteristics of malignant tumors (33, 36). Despite the authority of these persons, many other famous physicians took exception to such conclusions and considered a metastatic colloid goiter to be a benign tumor (33).

## Metastatic thyroid cancer at the beginning of the twentieth century and the concept of “benign metastatic goiter”

These two observations have had impressive significance for medicine generally and endocrinology specifically. The importance of these publications is two-fold. First, they suggested that thyroid cancer could metastasize, brought attention to the problem, and created a fruitful discussion in the scientific literature. The histological type of the carcinomas analyzed in these early reports was in general distinct from papillary thyroid cancer; it was probably follicular thyroid cancer. By 1880, at least five similar cases had been reported, and the number of publication was steadily increasing (37), which led to the conclusion that*”…the tumours occur most frequently in women (five to one) and are most common between the fortieth and sixtieth years. They show a striking preference for the skull but have been observed in the femur, clavicle, sternum, humerus, and on several occasions in the vertebrae” (* 37). Apparently, the lack of understanding of the various types of thyroid carcinomas and the classification of malignancies was the major obstacle. *“Here, it must be noted that the difficulty in discriminating between normal thyroid, goitrous thyroid, thyroid adenoma, and thyroid cancer is admitted by all experts to be very great … Even those who have had much experience in the microscopic examination of thyroid tumours will admit that it is often difficult to say where adenoma ends and carcinoma begins.”* (38).

Approximately at that time different histological types of thyroid cancer were established. In 1878, Theodor Billroth established papillary thyroid carcinoma as a subtype of the cancer (39). Later, in 1906, German Julius Jaquet described “malignant goiter with amyloid,” or medullary thyroid carcinoma (40). In 1907, another pathologist Theodor Langhans published a series of five cases now considered to represent the first description of Hürthle cell tumors (41). In 1879 Carl Kaufmann, a mentee of Kocher, summarized the knowledge on thyroid cancer in a 75-page key paper entitled “*Die Struma maligna. Primäres Sarkom und Carcinoma strumae”*, where he was the first to recommend the use of preoperative biopsy for the diagnosis of thyroid cancer (42). In 1883 Wölfler in suggested that most of thyroid tumors had their origin in fetal rests, which only began to grow in adult life (43). Presumably, at that time Wölfler recognized papillary microcarcinomas as “fetal nests”, but could not explain findings.

Secondly, in 1876, Julius Cohnheim introduced the term “*benign metastasizing goiter*,” but this term gives the impression of a completely benign morphology with no malignant findings (44). The concept of benign metastasizing goiter became popular in medical society. In 1923, the British Joll collected 44 cases “*in which one or more deposits in bone were found associated with what was considered either as a normal thyroid gland or a benign form of tumor or enlargement*” (35). Not all shared this opinion. The German pathologist Gierke believed that metastatic deposits in the bones were essentially malignant in spite of their apparently benign structure, even when the thyroid itself was normal (35). It required multiple studies, including those of Graham (1925), Simpson (1926), Levin (1930), Dinsmore and Hicken (1934) Friedman (1943), Outerbridge (1947), and Matovinovic (1971), to prove that the presence of metastases is a crucial indication of thyroid malignancy (32, 45–50).

The question of the function of metastatic tumors was raised by Gulliver in 1886 (51). (51) Iodine had been identified in metastatic deposits by Schnitzeler and Ewald (1896), Gierke (1902), and Meyer-Hürlimann and Oswald (1913) (52–54). The first attempt to investigate the metabolic function of thyroid metastases was performed by Meyer-Hürlimann and Oswald in 1913 (54). Twenty years later, Engelstad and, one year after that, Milles found considerable evidence that this tissue also has thyroid function (55, 56). These fundamental studies provided a strong background for the development of radioiodine for clinical use (57).

The concept of “*benign metastasizing goiter*” underestimated the significance and malignant potential of thyroid cancer. Once the debate surrounding that concept was concluded and fundamental and clinical research deepened our knowledge in thyroid cancer, endocrinology is now facing another opposite problem, a phenomenon commonly described as thyroid cancer “overdiagnosis” (58). The increasing incidence of thyroid carcinomas last decades is solely attributable to papillary thyroid carcinoma, more specifically papillary microcarcinomas. The debate on the appropriate treatment of patients with papillary thyroid cancer (PTC) has persisted for several decades and will likely to endure (59). The controversies regarding the optimal management of papillary thyroid microcarcinoma will likely to be addressed by future scientific breakthroughs in imaging, molecular prognostic markers that will help select individualized management (59).

## Conclusions

1. Max Runge and Julius Cohnheim’s observations made impressive significance for medicine generally and endocrinology as they showed that thyroid cancer could metastasize.
2. Cohnheim’s conception “*benign metastasizing goiter*” underestimated the significance and malignant potential of thyroid cancer and misled medical and endocrinological societies and complicate thyroid cancer research for several decades.
3. The problem of increasing incidence of thyroid carcinomas and the controversies regarding the optimal management of papillary thyroid microcarcinoma are other completely opposite issues, that endocrinology faces today. This story shows that dedicated researches and powerful science can address controversies and problems in previous studies and bring the best solutions into the clinical practice.

## Author contributions

SK: Conceptualization, Supervision, Visualization, Writing – original draft, Writing – review & editing. YK: Conceptualization, Writing – original draft, Writing – review & editing, Visualization. TG: Writing – review & editing. IP: Writing – review & editing. OT: Conceptualization, Writing – review & editing.
