# Corrigendum: Discovery of metastases in thyroid cancer and “benign metastasizing goiter”: a historical note

**DOI:** 10.3389/fendo.2024.1428680

**Published:** 2024-05-17

**Authors:** Sergiy Kushchayev, Yevgeniya Kushchayeva, Tetiana Glushko, Iryna Pestun, Oleg Teytelboym

**Affiliations:** ^1^ Department of Diagnostic Imaging and Interventional Radiology, Moffitt Cancer Center, Tampa, FL, United States; ^2^ Diabetes and Endocrinology Center, University of South Florida, Tampa, FL, United States; ^3^ Department of Radiology, Mercy Catholic Medical Center, Philadelphia, PA, United States

**Keywords:** cancer, thyroid, metastases, malignancies, goiter, history

In the published article, there was an error in the sequence of the authors’ affiliation(s) for Sergiy Kushchayev, Yevgeniya Kushchayeva, Tetiana Glushko, and Iryna Pestun.

Instead of “Sergiy Kushchayev **^1†^**, Yevgeniya Kushchayeva ^2*†^, Tetiana Glushko **^2^**, Iryna Pestun **^1^**

**^1^** Diabetes and Endocrinology Center, University of South Florida, Tampa, FL, United States

**^2^** Department of Radiology, Moffitt Cancer Center, Tampa, FL, United States”, the affiliations should be as follows.

Sergiy Kushchayev **^1†^**, Yevgeniya Kushchayeva ^2*†^, Tetiana Glushko **^1^**, Iryna Pestun **^2^**

**^1^** Department of Diagnostic Imaging and Interventional Radiology, Moffitt Cancer Center, Tampa, FL, United States

**^2^** Diabetes and Endocrinology Center, University of South Florida, Tampa, FL, United States.

The authors apologize for this error and state that this does not change the scientific conclusions of the article in any way.

